# Amphiregulin promotes cisplatin chemoresistance by upregulating ABCB1 expression in human chondrosarcoma

**DOI:** 10.18632/aging.103220

**Published:** 2020-05-19

**Authors:** Yu-Wen Huang, Chih-Yang Lin, Hsiao-Chi Tsai, Yi-Chin Fong, Chien-Kuo Han, Yuan-Li Huang, Wen-Tung Wu, Shih-Ping Cheng, Hao-Chiun Chang, Kuang-Wen Liao, Shih-Wei Wang, Chih-Hsin Tang

**Affiliations:** 1 Graduate Institute of Biomedical Sciences, China Medical University, Taichung, Taiwan; 2 Department of Medicine, Mackay Medical College, New Taipei, Taiwan; 3 Department of Medical Research, MacKay Memorial Hospital, Taipei, Taiwan; 4 Department of Sports Medicine, College of Health Care, China Medical University, Taichung, Taiwan; 5 Department of Orthopedic Surgery, China Medical University Hospital, Taichung, Taiwan; 6 Department of Biotechnology, College of Health Science, Asia University, Taichung, Taiwan; 7 Department of Food Science and Nutrition, Meiho University, Pingtung, Taiwan; 8 Department of Surgery, MacKay Memorial Hospital, Taipei, Taiwan; 9 Department of Orthopaedics, MacKey Memorial Hospital, Taipei, Taiwan; 10 Department of Pharmacology, School of Medicine, China Medical University, Taichung, Taiwan; 11 Chinese Medicine Research Center, China Medical University, Taichung, Taiwan; 12 Ph.D. Degree Program of Biomedical Science and Engineering, National Chiao Tung University, Hsinchu City, Taiwan; 13 Institute of Molecular Medicine and Bioengineering, National Chiao Tung University, Hsinchu, Taiwan; 14 Graduate Institute of Natural Products, College of Pharmacy, Kaohsiung Medical University, Kaohsiung, Taiwan

**Keywords:** chondrosarcoma, ABCB1, amphiregulin, chemotherapy, cisplatin

## Abstract

Chondrosarcomas are well known for their resistance to chemotherapeutic agents, including cisplatin, which is commonly used in chondrosarcomas. Amphiregulin (AR), a ligand of epidermal growth factor receptor (EGFR), plays an important role in drug resistance. We therefore sought to determine the role of AR in cisplatin chemoresistance. We found that AR inhibits cisplatin-induced cell apoptosis and promotes ATP-binding cassette subfamily B member 1 (ABCB1) expression, while knockdown of ABCB1 by small interfering RNA (siRNA) reverses these effects. High phosphoinositide 3-kinase (PI3K), Akt and nuclear factor kappa-light-chain-enhancer of activated B cells (NF-κB) phosphorylation levels were observed in cisplatin-resistant cells. Pretreating chondrosarcoma cells with PI3K, Akt and NF-κB inhibitors or transfecting the cells with p85, Akt and p65 siRNAs potentiated cisplatin-induced cytotoxicity. In a mouse xenograft model, knockdown of AR expression in chondrosarcoma cells increased the cytotoxic effects of cisplatin and also decreased tumor volume and weight. These results indicate that AR upregulates ABCB1 expression through the PI3K/Akt/NF-κB signaling pathway and thus contributes to cisplatin resistance in chondrosarcoma.

## INTRODUCTION

Chondrosarcoma is a malignant type of bone tumor consisting of transformed cells that produce the cartilage matrix [1]. Of the four major subtypes of chondrosarcoma – conventional, dedifferentiated, mesenchymal, clear cell, and periosteal [2] – conventional chondrosarcoma is the most common (~90%) [3] and is often curable through surgical treatment, especially when of low histologic grade [4]. However, different approaches are needed for unresectable disease, which typically manifests after multiple local recurrences or in patients with pulmonary metastases [5].

The identification of point mutations in isocitrate dehydrogenase-1 and isocitrate dehydrogenase-2 genes *IDH1* and *IDH2* in chondrosarcoma cell lines has prompted the development of specific agents that target these mutations, although the effectiveness and future role of such agents remains unclear [6]. Current treatment options for chondrosarcoma include chemotherapy followed by surgery and additional chemotherapy [7]. Patients with advanced disease and good performance status have reportedly derived clinical benefit with the palliative use of cisplatin and doxorubicin [4], although the relative resistance of chondrosarcomas to conventional chemo- and radiotherapy translates into very low 5- and 10-year survival rates [7].

Multiple mechanisms are responsible for the development of drug resistance. In particular, the adenosine triphosphate ATP-binding cassette subfamily B member 1 gene (*ABCB1*, also known as *MDR1*) [8], confers resistance to cytotoxic and targeted chemotherapy [9]. High levels of ABCB1 expression have been found in many different cancers, including thyroid, lung, and breast cancer, chronic myeloid leukemia, ependymoma, and osteosarcoma [10–15]; inhibiting ABCB1 expression reverses docetaxel resistance in prostate cancer [16], as well as paclitaxel and olaparib resistance in ovarian cancer cells [17]. Few studies have investigated the role of ABCB1 in chondrosarcoma.

Amphiregulin (AR), an epidermal growth factor that is a ligand of epidermal growth factor receptor (EGFR) [18], has been identified in various physiological processes, including the regulation of lung morphogenesis, mammary gland development, bone formation, cell invasiveness and angiogenesis [19–21]. Notably, high AR expression is associated with worse survival outcomes in several different cancers, including ovarian, glioma, head and neck, breast, and lung cancers [22–24].

In this study, we identified higher levels of AR and ABCB1 expression in cisplatin-resistant human chondrosarcoma SW1353 cells (cis-SW) compared with cisplatin-sensitive SW1353 cells. Inhibition of AR sensitized chondrosarcoma cells to cisplatin in cellular and preclinical investigations, indicating that AR is a promising target in cisplatin-resistant human chondrosarcoma.

## RESULTS

### Amphiregulin contributes to cisplatin resistance in human chondrosarcoma cells

**Figure 1:**
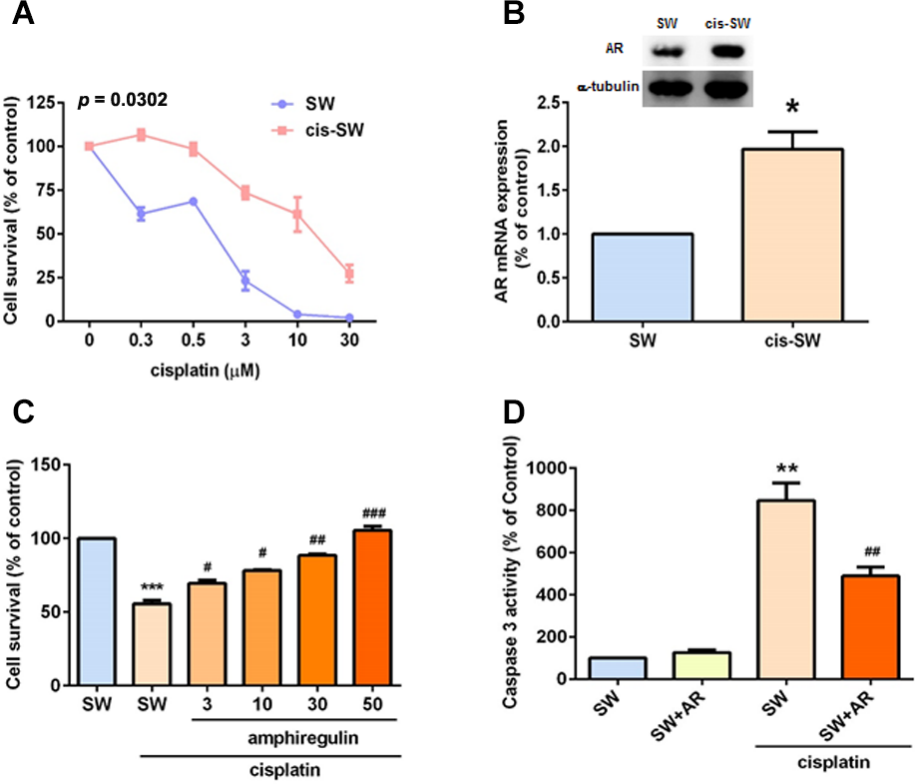
**Amphiregulin is involved in cisplatin resistance in human chondrosarcoma cells.** (**A**) SW (SW1353) and cis-SW (cisplatin-resistant) cells were treated with different concentrations of cisplatin for 24 h and cell viability was analyzed using the MTT assay. (**B**) Intracellular AR levels in whole cell lysates were analyzed by Western blot and qPCR assays. (**C**) Chondrosarcoma cells were incubated with various concentrations of AR for 24 h. Cell viability was examined by MTT assay. (**D**) Chondrosarcoma cells were treated with cisplatin (1 μM) for 24 h and cell apoptosis was studied according to levels of caspase-3 activity. The results were obtained from 3 independent experiments and are expressed as the mean ± SEM. * *p* < 0.05; ** *p* < 0.01; *** *p* < 0.001 compared with controls; ^#^ *p* < 0.05; ^##^ *p* < 0.01; ^###^ *p* < 0.001 compared with cisplatin-treated controls.

Tumor progression involves members of the EGF/neuregulin family [22], including the EGFR ligand, AR. In several cancers (e.g., ovarian, glioma, head and neck, breast, and lung cancers), high AR expression correlates with worse prognosis [22–24]. In our previous study, we reported that AR enhances chondrosarcoma cell migration and resistance to doxorubicin [25]. We therefore hypothesized that AR may play a role in cisplatin resistance in the treatment of chondrosarcoma. We used the human chondrosarcoma cell line SW1353 to establish cisplatin-resistant cells (cis-SW), which exhibited high levels of survival and AR expression (Figure 1A, 1B). We then pretreated chondrosarcoma cell lines JJ012 and SW1353 with different concentrations of exogenous recombinant AR and found that AR effectively and dose-dependently promoted cell viability in the presence of cisplatin (Figure 1C and Supplementary Figure 1), confirming that AR increases levels of cisplatin resistance in JJ012 and SW1353 cells. Moreover, cisplatin-mediated cell apoptosis was decreased after AR treatment (Figure 1D), according to levels of caspase-3 activity. These data confirm that AR increases levels of cisplatin resistance in chondrosarcoma cells, by inhibiting cisplatin-induced cell apoptosis.

### Knockdown of amphiregulin expression suppresses cisplatin resistance in human chondrosarcoma cells

**Figure 2:**
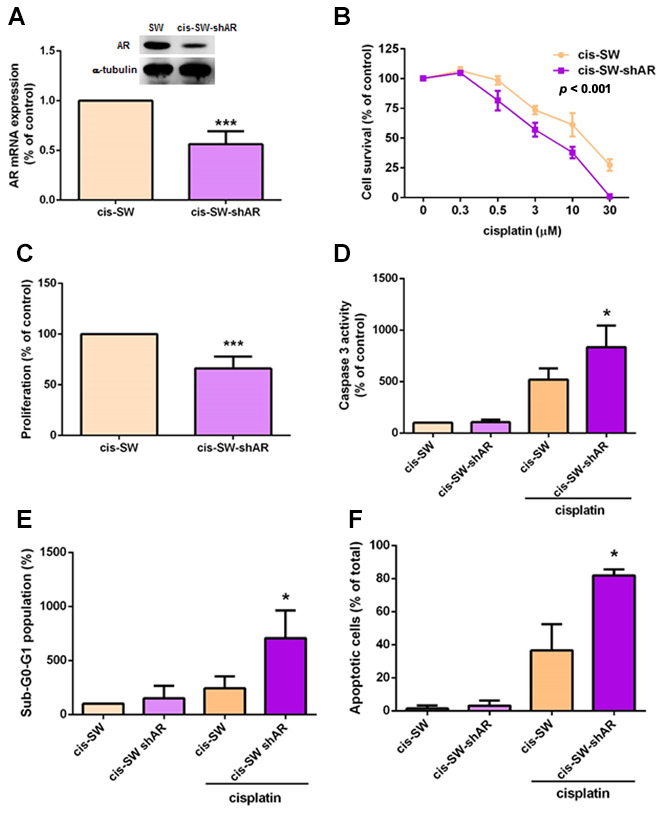
**Knockdown of amphiregulin expression suppresses cisplatin resistance in human chondrosarcoma cells.** (**A**) Intracellular AR levels in whole cell lysates were analyzed by Western blot and qPCR assays. (**B**) Chondrosarcoma cells were treated with different concentrations of cisplatin for 24 h and cell viability was analyzed using the MTT assay. (**C**) Cell proliferation rates were determined by the MTT assay. (**D**–**F**) Chondrosarcoma cells were treated with cisplatin (10 μM) for 24 h and cell apoptosis was examined by caspase-3 activity (**D**), PI staining (**E**), and Annexin V-FITC binding. (**F**) The results were obtained from 3 independent experiments and are expressed as the mean ± SEM. * *p* < 0.05; ** *p* < 0.01; *** *p* < 0.001 compared with controls.

In an attempt to further clarify the role of AR in cisplatin resistance in chondrosarcoma cells, we transfected cis-SW cells with lentivirus expressing AR shRNA (cis-SW-shAR) and Western blot as well as qPCR assays confirmed potent knockdown of AR expression (Figure 2A), with significant inhibition of cell viability and proliferation (Figure 2B, 2C). Furthermore, decreased AR expression promoted cisplatin-induced apoptosis (Figure 2D–2F). These data demonstrate that AR promotes cisplatin resistance in chondrosarcoma cells.

### ABCB1 is involved in amphiregulin-mediated chemoresistance

**Figure 3:**
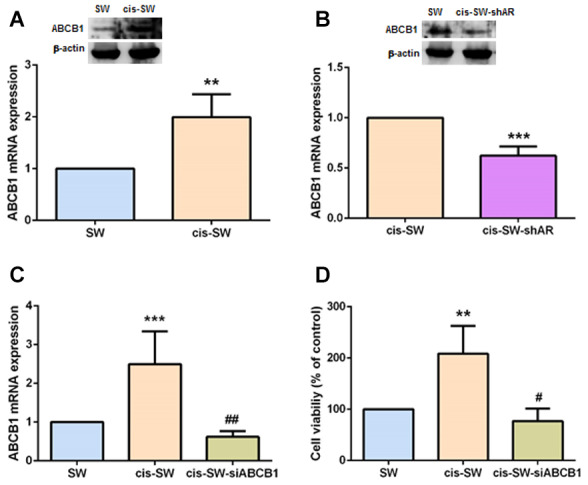
**ABCB1 is involved in amphiregulin-mediated chemoresistance.** (**A**, **B**) Levels of ABCB1 gene and protein expression in chondrosarcoma cells were detected by qPCR and Western blot assays. (**C**) Cis-SW cells were transfected with ABCB1 siRNA, and ABCB1 mRNA expression was examined by qPCR assay. (**D**) Cis-SW cells were transfected with ABCB1 siRNA, then treated with cisplatin (10 μM) for 24 h. Cell viability was examined by MTT assay. The results were obtained from 3 independent experiments and are expressed as the mean ± SEM. * *p* < 0.05; ** *p* < 0.01; *** *p* < 0.001 compared with controls; ^#^ *p* < 0.05; ^##^ *p* < 0.01; ^###^ *p* < 0.001 compared with cisplatin-treated controls.

ABCB1 confers a multidrug-resistant phenotype in cancers, limiting the absorption and toxicity of chemotherapeutic agents [9]. We therefore speculated that ABCB1 expression correlates with levels of cisplatin resistance in chondrosarcoma cells. As shown in Figure 3A, cis-SW cells expressed high levels of ABCB1 expression, which were significantly decreased when the cells were transfected with lentivirus expressing AR shRNA, as determined by Western blot and qPCR assays (Figure 3B). Furthermore, when we transfected ABCB1 small interfering RNA (siRNA) into cis-SW cells, we observed a significant decrease in levels of ABCB1 mRNA expression (Figure 3C) and a significant decrease in cell viability (Figure 3D). Thus, ABCB1 plays an important role in AR-induced cisplatin resistance in human chondrosarcoma cells.

### Amphiregulin activates PI3K, Akt, and NF-κB signaling pathways during chemoresistance

**Figure 4:**
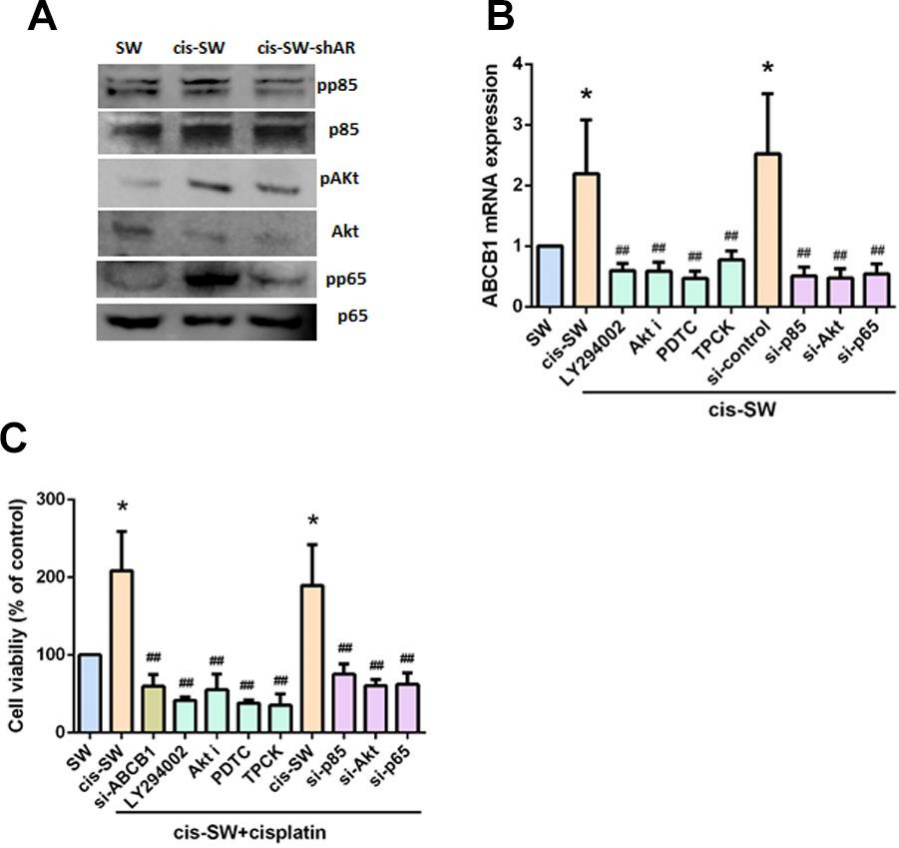
**Amphiregulin contributes to chemoresistance by activating the PI3K, Akt, and NF-kB signaling pathways.** (**A**) Protein expression was examined by Western blot assay. (**B**, **C**) Cells were pretreated with a PI3K inhibitor (Ly294002, 10uM), an Akt inhibitor (Akt i, 10 mM), or an NF-κB inhibitor (PDTC, 10 mM; TPCK, 3uM) or transfected with p85, Akt, p65, or ABCB1 siRNA, followed by stimulation with cisplatin for 24 h. Levels of ABCB1 expression and cell viability were detected by qPCR and MTT assays. The results were obtained from 3 independent experiments and are expressed as the mean ± SEM. * *p* < 0.05; ** *p* < 0.01; *** *p* < 0.001 compared with controls; ^#^ *p* < 0.05; ^##^ *p* < 0.01; ^###^ *p* < 0.001 compared with cisplatin-treated controls.

Phosphatidylinositol 3-kinase (PI3K) signaling stimulates cancer cell growth and survival, motility and metabolism [26]. The PI3K, Akt, and nuclear factor kappa-light-chain-enhancer of activated B cells (NF-κB) signaling cascade is one of the main canonical pathways implicated in cancer pathogenesis, including chemoresistance [27–29]. We therefore examined whether the PI3K/Akt/NF-κB pathway is involved in AR-mediated ABCB1 expression and chemoresistance. We observed increased levels of PI3K, Akt and NF-κB phosphorylation in cis-SW cells and decreased levels in cis-SW-shAR cells, compared with levels in SW1353 cells (Figure 4A). Pretreatment of cis-SW cells with a PI3K inhibitor (Ly294002), an Akt inhibitor (Akt i), or NF-κB inhibitors (PDTC and TPCK), or transfection with p85, Akt, p65, and ABCB1 siRNAs decreased AR-mediated ABCB1 expression and cell viability (Figure 4B, 4C), suggesting that the PI3K, Akt, and NF-κB signaling pathways mediated AR-increased resistance to cisplatin.

### Inhibiting amphiregulin expression suppresses *in vivo* resistance to cisplatin

**Figure 5:**
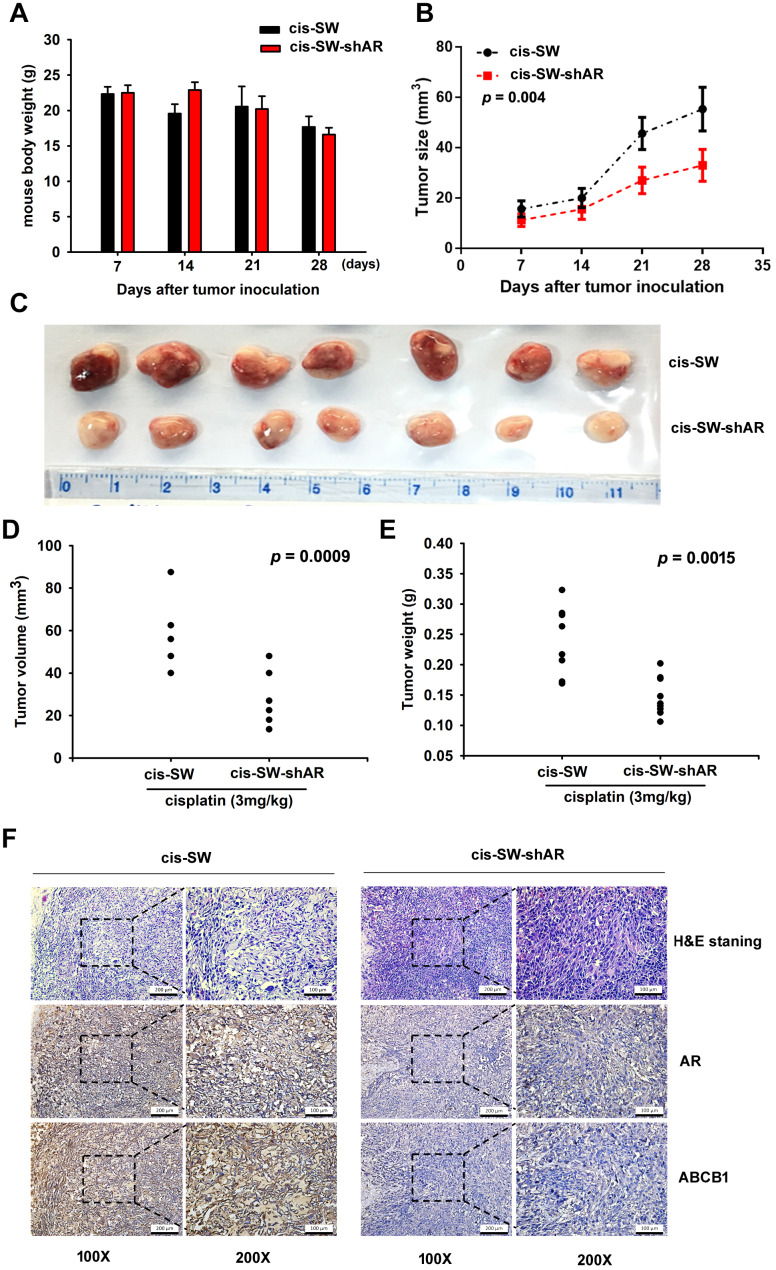
**Inhibiting amphiregulin expression suppresses resistance to cisplatin in a nude mouse xenograft model.** (**A**) Body weights are shown for mice treated with cisplatin for 28 days. (**B**) Tumor growth curves of chondrosarcoma cells treated with cisplatin over 28 days. (**C**) Representative photomicrographs of cis-SW and cis-SW-shAR cells from nude mice. (**D**, **E**) Tumor volumes and weights were measured after the mice were sacrificed. (**F**) IHC staining detected AR and ABCB1 expression.

We next investigated the *in vivo* role of AR, by injecting cis-SW and cis-SW-shAR cells into the flanks of nude mice. We found that AR knockdown did not affect the weight of the mice (Figure 5A), but did increase the cytotoxic effects of cisplatin over the 28-day observation period (Figure 5B). As shown in Figures 5C and 5D, tumor volumes and weight were lower in the mice administered AR knockdown compared with those that were not. Finally, IHC staining revealed substantially reduced levels of AR and ABCB1 expression in the cis-SW-shAR group compared with the cis-SW group (Figure 5E). Our data indicate that AR knockdown suppresses drug resistance and the *in vivo* evidence supports our hypothesis that AR acts as an oncogene, inhibiting chemotherapeutic activity in human chondrosarcoma.

## DISCUSSION

Treatment of patients with chondrosarcoma is complicated by the significant issues of lung metastasis, tumor recurrence and multidrug resistance [2]. The identification of reliable biomarkers and targetable molecules would greatly assist with the monitoring of disease progression and enable clinicians to administer timely treatment for patients with chondrosarcoma [30]. The presence of AR is closely linked to the oncogenic process; increasingly higher levels of AR expression correlate with worse prognosis in several cancers, such as ovarian, glioma, head and neck, breast, and lung cancers [22–24]. We have previously reported that AR enhances resistance to doxorubicin chemotherapy, apparently by enhancing chondrosarcoma cell migration and resistance by activating the MAPK pathway [25]. Evidence in that study strongly suggested that inhibiting MAPK activation successfully blocks cell migration and that doxorubicin sensitivity is improved by inhibiting p38 activation and increasing ERK activation [34]. Here, we have characterized how AR contributes to the development of cisplatin resistance in chondrosarcoma. The findings from our studies yield important insights into how we might improve the outcome for patients with chondrosarcoma, for which current treatment options have limited therapeutic efficacy and conventional cytotoxic chemotherapy has generally been thought to be ineffective [7].

Chemotherapeutic drug resistance involves multiple mechanisms; the dysregulation of ABC membrane transporters is one of the most important [31]. High levels of ABCB1expression are observed in various cancers resistant to paclitaxel [17]. Moreover, many cancers are characterized by overexpression of ABCB1, including thyroid cancer, lung cancer, breast cancer, chronic myeloid leukemia, ependymoma, and osteosarcoma [10–15]. Some researchers have speculated that the activity of ABCB1 pumps, which cause the efflux of chemotherapeutic agents in various cancers, may be associated with resistance to doxorubicin in chondrosarcoma [32]. This hypothesis is supported by the findings of our present study, in which the upregulation of ABCB1 expression in a cisplatin-resistant cell line (cis-SW) promoted cell viability in the presence of cisplatin. Thus, our findings highlight the important role of ABCB1 in cisplatin resistance in chondrosarcoma.

PI3K signaling stimulates cancer cell growth and survival, motility and metabolism [26]. Blocking the PI3K/Akt/NF-κB signaling pathway reverses oxaliplatin resistance in the treatment of colorectal cancer [33], while phosphorylation of Akt by PI3K induces drug resistance in various tumor types [34]. Akt also contributes to drug resistance in cancer cells by phosphorylating nuclear transcription factor NF-κB, activating several survival genes [35]. PI3k, Akt, and NF-κB activation is involved in ABCB1 expression and cell death [33, 36–38]. These observations are supported by our study evidence revealing high levels of PI3k, Akt, and NF-κB phosphorylation in cis-SW cells and we have demonstrated that AR-induced increases in levels of ABCB1 expression and resistance to cisplatin were reversed by treatment with PI3k, Akt and NF-κB inhibitors.

**Figure 6:**
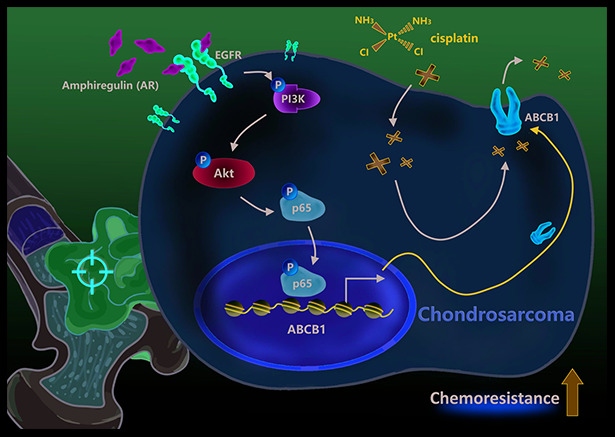
**Schematic presentation of the signaling pathways involved in amphiregulin-mediated chemoresistance in human chondrosarcoma cells.**

New treatment options are urgently needed for chondrosarcoma, especially for inoperable or metastatic disease [7]. Our evidence suggests that the targeting of AR has therapeutic potential, as AR inhibited cisplatin-induced apoptosis in chondrosarcoma cells. High levels of ABCB1 expression exhibited by cis-SW cells were significantly decreased by AR knockdown. We also found high levels of PI3K, Akt and NF-κB phosphorylation in cis-SW cells and decreased levels in cis-SW-shAR cells, compared with SW1353 cells. Pretreatment of cells with PI3K, Akt, and NF-κB inhibitors or transfection with p85, Akt, p65, and ABCB1 siRNAs inhibited AR-induced increases in ABCB1 expression and cell viability. Our results suggest that the PI3K/Akt/NF-κB signaling pathway mediates AR-mediated resistance to cisplatin in chondrosarcoma (Figure 6).

These *in vitro* results were supported by our preclinical findings, in which the inhibition of AR expression increased cisplatin-induced cytotoxicity and decreased tumor volumes and weights. AR appears to be a critical oncogene that confers chemoresistance in chondrosarcoma cells. We believe that our data support an investigation into the targeting of AR with neutralizing antibodies, to better determine whether such a strategy constitutes an effective approach to chondrosarcoma.

## CONCLUSIONS

In this study, we identified high levels of AR and ABCB1 expression in human chondrosarcoma cisplatin-resistant cells. AR upregulates ABCB1 expression through the PI3K/Akt/NF-κB signaling pathway, and knockdown of AR sensitized chondrosarcoma cells to cisplatin in cellular and preclinical experiments. AR is therefore worth pursuing as a therapeutic target in cisplatin-resistant human chondrosarcoma.

## MATERIALS AND METHODS

### Materials

Human recombinant amphiregulin protein was purchased from PeproTech (Rocky Hill, NJ, USA). Anti-rabbit and anti-mouse IgG-conjugated horseradish peroxidase, rabbit polyclonal antibodies specific for pAkt (Catalog No. sc-16646) and ABCB1 (Catalog No. sc-55510), and mouse monoclonal antibodies specific for p85 (Catalog No. sc-1637), Akt (Catalog No. sc-5298) and p65 (Catalog No. sc-8008) were purchased from Santa Cruz Biotechnology (Santa Cruz, CA, USA). Antibodies for pp85 (Catalog No. 4228S) and pp65 (Catalog No. 3033) were purchased from Cell Signaling Technology (Santa Cruz, CA, USA). DharmaFECT transfection reagents (Catalog ID: T-2010-01), small interfering RNAs (siRNAs) against ABCB1 (Catalog No. L-003868-00-0005), p85 (Catalog No. L-003020-00-0005), Akt (Catalog No. L-003000-00-0005) and p65 (Catalog No. L-003533-00-0005) were purchased from Dharmacon Research (Lafayette, CO, USA). Ly294002 (Catalog No. ALX-270-038) was purchased from Enzo Life Sciences (Farmingdale, New York, USA). Akt inhibitor (Akt i) (Catalog No. A6730), TPCK (Catalog No. T4376-100MG), PDTC (Catalog No. P8765-1G) and all other chemicals were purchased from Sigma-Aldrich (St. Louis, MO, USA).

### Cell culture

The human chondrosarcoma cell line SW1353 was obtained from the American Type Cell Culture Collection (Manassas, VA, USA), and JJ012 was kindly supplied by Dr. Sean P. Scully (University of Miami School of Medicine). Chondrosarcoma cell culture conditions were prepared according to a previously described protocol [39].

### Establishment of a cisplatin-resistant cell line

A cisplatin-resistant cell line was established using SW1353 cells subjected to stepwise increments of cisplatin concentrations in culture medium, according to the methodology from a previous report [40]. When the cells reached 60–70% confluency in the culture dish, cisplatin was added to the cells at concentrations of 0.1, 0.2, 0.5, or 1 μM. After 2 days of continuous exposure to cisplatin, the medium was replaced with fresh cisplatin-free medium until the surviving cells showed favorable recovery rates. After the cells had recovered their previous confluency rates (60–70%), cisplatin was added again to the medium, and each concentration experiment was repeated 3 times. After 6 months, cells that grew in the medium with 1 μM of cisplatin were designated as cis-SW cells and stored for further investigation.

### The 3-(4,5-dimethylthiazol-2-yl)-2,5-diphenyltetrazolium bromide (MTT) assay

Cell viability was determined by the MTT assay, according to the methods used in our previous studies [41–43]. Cells were plated at a concentration of 5,000 cells per well in 96-well plates. After cisplatin treatment, cultures were washed with PBS and 0.5 mg/ml of MTT solution was added, before the mixture was incubated at 37°C for 1 h. The formazan crystals were dissolved in DMSO and the absorbance was determined at 550 nm.

### Quantitative real-time PCR

The StepOnePlus sequence detection system used for qPCR assays was performed according to an established protocol [44–46]. Total RNA was extracted from chondrosarcoma cells using the TRIzol kit (Catalog No. 15596026) (MDBio, Taipei, Taiwan) and cDNA was synthesized using the M-MLV Reverse Transcriptase kit (Catalog No. 28-025-013) (Invitrogen, Carlsbad, CA, USA). 100 ng of total cDNA was mixed with sequence-specific primers using the KAPA SYBR^®^ FAST qPCR Kit (Catalog No. KK4601) (Applied Biosystems, Foster City, CA, USA). The cycling conditions were as follows: polymerase activation for 10 min at 95°C followed by 40 cycles at 95°C for 15 s and 60°C for 1 min. Relative normalization of gene expression was performed using endogenous glyceraldehyde 3-phosphate dehydrogenase (GAPDH) as the internal control. Primer sequences were as follows: amphiregulin forward primer: 5’-GTGGTGCTGTCGCTCTTGATA-3’; amphiregulin reverse primer: 5’-CCCCAGAAAATGGTTCACGCT-3’; ABCB1 forward primer: 5’-TTGCTGCTTACATTCAGGTTTCA-3’; ABCB1 reverse primer: 5’- AGCCTATCTCCTGTCGCATTA-3’; GAPDH forward primer: 5’-ACCACAGTCCATGCCATCAC-3’; GAPDH reverse primer: 5’-TCCACCACCCTGTTGCTGTA-3’.

### siRNA transient transfection

Chondrosarcoma cells (1 × 10^5^ cells/well) were seeded into 12-well plates. The following day, the cells were transfected for 24 h with 100 nM of ON-TARGETplus siRNAs using DharmaFECT transfection reagent (Catalog ID: T-2010-01). After 24 h, cells were collected and stored for further investigation.

### Western blot analysis

Cell lysates were prepared by RIPA buffer containing a protease inhibitor cocktail and the concentration of protein was determined using the BCA Protein Assay Kit (Catalog No. 23225) (Thermo Fisher Scientific Inc., Rockford, IL, USA). Proteins were resolved with SDS-PAGE and transferred to Immobilon^®^ polyvinylidene difluoride (PVDF) membranes. The blots were blocked at room temperature using 5% BSA for 1 h, then incubated with primary antibodies (1:3000) for an additional 1 h. After undergoing 3 washes in TBST buffer (0.05% Tween 20 in Tris-buffered saline), the blots were subsequently incubated with secondary antibody. The bands were visualized using ImageQuant™ LAS 4000 (GE Healthcare, Little Chalfont, UK) [47, 48].

### Enzymatic assay for caspase-3 activity

Caspase-3 activity in cell lysates was assayed using the Ac-DEVD-pNA colorimetric substrate for caspase-3, according to an established protocol [41]. An equal amount of total protein extract was incubated overnight with Ac-DEVD-pNA at 37°C. The release of *p*-nitroaniline (pNA) was monitored at 405 nm using a microplate ELISA reader (Bio-Rad Laboratories, Inc.). Results are presented with the percentage change in activity compared with the untreated controls.

### Quantification of apoptosis by flow cytometry

A total of 3 × 10^6^ cells/ml was infused with 0.5 ml of pre-chilled ethanol, then incubated for 30 min at 4°C. The ethanol was then removed and cellular DNA was stained with 100 μg/ml propidium iodide (PI) with 100 μg/ml of DNase-free RNase. Cellular apoptosis was quantified by the amount of fractional DNA content in the sub-G_1_ peak phase of the cell cycle.

The Annexin V/fluorescein isothiocyanate (FITC) apoptosis detection kit II (Catalog No. BD 556570) (BD Biosciences, Pharmingen) was used to assess the binding of Annexin V-FITC to phosphotidylserine, according to the manufacturer’s protocol. Briefly, cells were resuspended in the binding buffer and reacted with 5 μl of Annexin V-FITC reagent and 5 μl of propidium iodide in the dark for 30 min at room temperature. Stained cells were analyzed by fluorescent-activated cell sorting (FACS) on a FACScan flow cytometer (Becton Dickinson).

### Murine xenograft experiments

To generate murine subcutaneous tumors, 6-week-old male BALB/c nude mice were randomly divided into 2 groups (10 mice per group). 1 × 10^6^ chondrosarcoma cells were injected subcutaneously into the right flanks of mice (purchased from the National Science Council Animal Center, Taipei, Taiwan). Four weeks after injection, the subcutaneous tumor size had reached a diameter of approximately 1,000 mm^3^, and the mice received intraperitoneal (i.p.) injections of cisplatin (3 mg/kg) twice a week thereafter. Mouse weight and tumor volumes were calculated weekly. After 28 days, mice were euthanized by CO_2_ inhalation. Tumors were removed and photographed, fixed in 10% formalin and embedded in paraffin, then subjected to immunohistochemistry (IHC) staining. All mice were handled in accordance with the Animal Care and Use Guidelines of the China Medical University (Taichung, Taiwan), under a protocol approved by the Institutional Animal Care and Use Committee (IACUC).

### Immunohistochemistry staining

Tumor cell sections were deparaffinized with xylene and rehydrated with ethanol. The NovoLink Polymer System (Catalog No. RE7150-CE) (Leica Microsystems) was used to perform IHC staining according to the manufacturer’s protocol. Human AR or ABCB1 antibody was applied at a dilution of 1:200 then incubated at 4°C overnight. The sections were counterstained with hematoxylin. IHC results were scored by accounting for the percentage of positive detection and intensity of the staining in calculations using Image J software [49, 50].

### Statistics

Statistical data were analyzed using SPSS Statistics version 20.0 (SPSS, Chicago, USA). All data are expressed as the mean ± standard error of the mean (SEM). Statistical comparisons between two samples were performed using the Student’s *t*-test, and a one-way analysis of variance (ANOVA) with *post hoc* Bonferroni correction was used to compare multiple groups. A p-value of < 0.05 was considered significant.

## Supplementary Material

Supplementary Figure 1
