# Supplementary material for: Amphiregulin promotes cisplatin chemoresistance by upregulating ABCB1 expression in human chondrosarcoma
Source: Aging (Albany NY). 2020 May 19;12(10):9475–88. doi: 10.18632/aging.103220 (PMC7288968; doi:10.18632/aging.103220)
Supplement: Supplementary Figure 1 [file aging-12-103220-s001..pdf]

SUPPLEMENTARY FIGURE

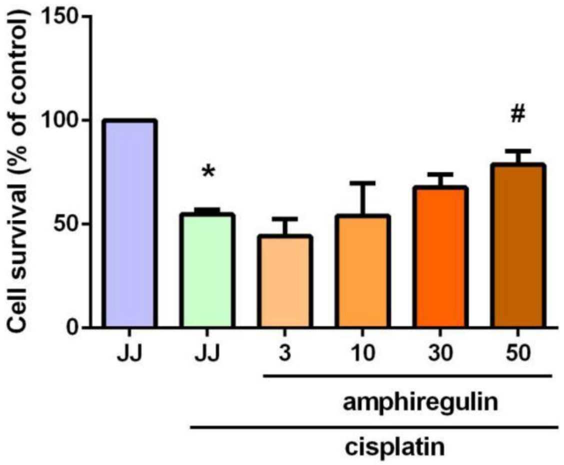

Supplementary Figure 1. Amphiregulin is involved in cisplatin resistance in human chondrosarcoma cells. Chondrosarcoma cell line (JJ012 cell) was treated with various concentrations of exogenous recombinant amphiregulin (AR) for 48 h, and then treated cisplatin (30uM) for another 24 h. Cell viability was examined by MTT assay. * $p < 0.05$; $^{\#}$ $p < 0.05$ compared with cisplatin-treated controls.
